# Unlocking the Bioactivity of Sweet Wormwood (*Artemisia annua* L., Asteraceae) Ethanolic Extract: Phenolics, Antioxidants, and Cytotoxic Effects

**DOI:** 10.3390/pharmaceutics17070890

**Published:** 2025-07-09

**Authors:** Neda Gavarić, Milica Aćimović, Nebojša Kladar, Maja Hitl, Jovana Drljača Lero, Nataša Milić, Katarina Radovanović

**Affiliations:** 1 Department of Pharmacy, Faculty of Medicine, University of Novi Sad, Hajduk Veljkova 3, 21000 Novi Sad, Serbia; nebojsa.kladar@mf.uns.ac.rs (N.K.); maja.bekut@mf.uns.ac.rs (M.H.); jovana.drljaca-lero@mf.uns.ac.rs (J.D.L.); natasa.milic@mf.uns.ac.rs (N.M.); katarina.radovanovic@mf.uns.ac.rs (K.R.); 2 Institute of Field and Vegetable Crops Novi Sad, National Institute of the Republic of Serbia, Maksima Gorkog 30, 21000 Novi Sad, Serbia; milica.acimovic@ifvcns.ns.ac.rs

**Keywords:** sweet wormwood, chlorogenic acid, antioxidant activity, cytotoxicity, cell viability

## Abstract

**Objectives**: The aim of this work was to determine the phenolic composition of sweet wormwood (*Artemisia annua* L., Asteraceae) from controlled cultivation in Serbia and to assess the potential antioxidant effects and cytotoxicity. **Methods**: High-performance liquid chromatography was used to determine the phenolic composition of *Artemisia annua* ethanolic extract. The antioxidant activity was studied using in vitro tests of inhibition of the neutralization of 2,2-diphenyl-1-picrylhydrazyl (DPPH), hydroxyl (OH), and nitroso (NO) radicals, as well as the process of inhibiting lipid peroxidation and the ferric reducing antioxidant power (FRAP). The cytotoxicity was evaluated by the effect on three cell lines (the rat pancreatic insulinoma cell line (Rin-5F), the rat hepatoma cell line (H4IIE), and human hepatocellular carcinoma (Hep G2)) using the MTT test of viability. **Results**: Ethanol extract showed the highest potency in inhibiting the DPPH radical, and the half maximal inhibitory concentration (IC_50_) was 5.17 μg/mL. Chlorogenic acid was the dominant phenolic compound with an amount of 651 μg/g of dry extract. The results of the MTT viability test showed that the extract has the potential to inhibit the growth of the Rin-5F and Hep G2 cell lines, while no growth inhibition was observed on the H4IIE cell line. **Conclusions**: Undoubtedly, *Artemisia annua* is a powerful plant and a rich source of phenolic compounds. Inhibitory activity on causes of oxidative stress shows that the plant has a good antioxidant effect. Also, the anticancer activity shown through the inhibition of cell growth is not negligible.

## 1. Introduction

Sweet wormwood (*Artemisia annua* L.) is an aromatic annual herb belonging to the genus *Artemisia* within the family Asteraceae [1]. This annual plant is native to temperate regions of Asia and is now cultivated all over the world, particularly in Central, Southeastern, and Southern Europe, as well as North America [2,3]. Dried aerial parts of *Artemisia annua* have been used for centuries in Traditional Chinese Medicine (TCM). As a part of the Chinese pharmacopeia, *Artemisia annua* is recognized as a remedy for malaria, fever caused by tuberculosis, jaundice, and other types of fever. In TCM, preparations such as aqueous decoctions or water extracts, infusions in cold water, or powdered herbs are primarily used [4]. The whole flowering plant has anthelmintic, antipyretic, antiseptic, antispasmodic, carminative, stimulant, tonic, and stomachic properties, and its crushed dry leaves have been used to treat diarrhea [4]. Although *Artemisia annua* herb preparations have a long tradition of use, the plant only gained global attention in the 1970s with the discovery of artemisinin, a sesquiterpene lactone extracted from its leaves [4].

*Artemisia annua* produces a wide range of secondary metabolites, broadly classified as volatile and non-volatile components. The volatile fraction consists mainly of the essential oil components, comprising up to 0.35% of the plant’s mass [5]. Major compounds, approximately 70% of the essential oil, are mono- and sesquiterpenoids such as camphene, β-camphene, isoartemisia ketone, 1-camphor, β-caryophyllene, and β-pinene. Other minor volatiles include artemisia ketone, 1,8-cineole, camphene hydrate, and cuminal [4,6,7]. Depending on the geographical origin of the plant, the differences in the percentage content of the main components of the essential oil occur [8]. The main non-volatile constituents include phenolic compounds such as phenolic acids, flavonoids, and coumarins, as well as terpenoids, including sesquiterpene lactones and steroids [9,10]. Sesquiterpene lactones are considered the primary bioactive constituents of *Artemisia annua,* with over thirty identified in the plant. Among them, artemisinin is the most often studied and pharmacologically significant [7,11,12].

Nowadays, various extracts of *Artemisia annua* are being extensively studied in scientific research, focusing on the isolation of active constituents, optimizing the extraction process, and therapeutic efficacy. Most often, organic solvent extracts are used since they have proven to be suitable for not only artemisinin extraction but also the extraction of a broader spectrum of bioactive compounds. Among the investigated organic solvents, ethanol is often used for *Artemisia annua* and other medicinal plant extractions, as it has several key advantages. For example, 70% ethanol possesses optimal polarity for broad-spectrum extraction of both polar (flavonoids, phenolic acids, glycosides) and moderately non-polar compounds (artemisinin, terpenoids) and can penetrate plant cell walls more effectively than water. Moreover, cold or room-temperature ethanol extraction preserves heat-sensitive compounds such as artemisinin and certain flavonoids [13]. Also, the antimicrobial and preservative properties of 70% ethanol reduce the microbial contamination of the extract and the potential microbial degradation of the chemical constituents. It is generally recognized as safe and suitable for herbal drug preparations that could be used in therapy. Broad-spectrum extraction contributes to the variety of chemical constituents in the final product, which may enhance its pharmacological activity due to the possible synergistic effects of different compounds [13,14]. The plant’s antioxidant potential is attributed to its high content and the diversity of these phenolic acids and flavonoids [7,11,12,15,16,17,18]. The antioxidant activity of various *Artemisia annua* extracts may be beneficial in preventing or reducing diseases associated with oxidative stress. Modern pharmacological studies have confirmed its potential anticancer, antiviral, anti-inflammatory, antifungal, antibacterial, immunomodulatory, and antiparasitic activities [2,12].

Due to its medicinal importance, there is a growing interest in the cultivation of *Artemisia annua* for pharmaceutical purposes to meet the increasing demand for artemisinin. The current research focuses on improving crop yield and quality through agricultural practices, tissue culture, biotechnological methods, and sustainable production. Moreover, for both ecological and economic reasons, it is important to valorize all potentially pharmacologically active constituents of *Artemisia annua*, namely phenolic compounds with strong antioxidant potential [19].

All things considered, there is no doubt that this powerful plant has a wide range of applications in the prevention and treatment of various health conditions. For the safe and effective use of *Artemisia annua* in modern medicine, comprehensive research on its phytochemical composition, pharmacological properties, and safety is required. Also, it is important to collect as much in vitro data as possible in order to be able to design clinical trials targeting the diverse therapeutic applications of *Artemisia annua* and its bioactive constituents. Therefore, the aim of this study was to determine the chemical composition of ethanol extract of *Artemisia annua*, cultivated under controlled conditions in Serbia, and to assess its potential antioxidant and cytotoxic activity.

## 2. Materials and Methods

### 2.1. Plant Material

**Figure 1:**
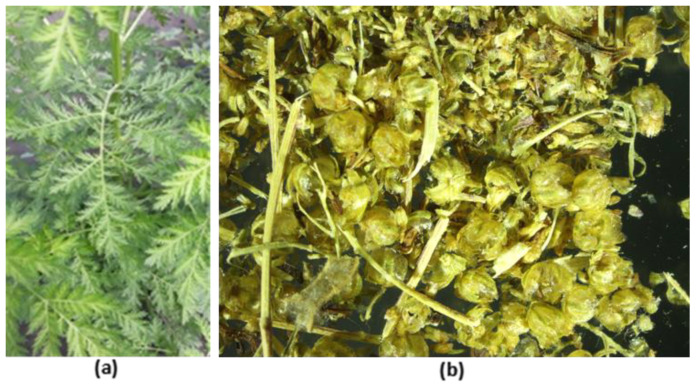
*Artemisia annua*: (**a**) leaves in the vegetative phase; (**b**) herbal drug prepared for the extraction consisting of the fragmented inflorescence, leaves, and stems.

The aerial parts of sweet wormwood (*Artemisia annua* L., Asteraceae) (Figure 1) at a full flowering stage were used for this study. The plant material was collected in June 2019 from the botanical garden of the Institute of Field and Vegetable Crops Novi Sad (IFVCNS), Serbia (Department of Vegetable and Alternative Crops Bački Petrovac). After collection, the plant material was air-dried in the shade to constant weight and then stored in multilayer paper bags under dry conditions until further analysis. The botanical identity of the plant was confirmed, and the voucher samples (No. 2-1513) were deposited in the Herbarium of the Department of Biology and Ecology (BUNS Herbarium), Faculty of Science, University of Novi Sad. Dried plant material was ground using an electric mill, in pulse mode, to prevent overheating of the herbal material and to preserve the integrity of the bioactive compounds.

### 2.2. Extract Preparation and Extraction Yield Determination

Ethanolic extract of *Artemisia annua* herb was prepared using 70% ethanol (*v*/*v*) as the extraction solvent. Maceration was performed for 24 h at a drug-to-solvent ratio of 1:10 (*w*/*w*). The obtained extract was then filtered and the solvent was evaporated to dryness. The extraction yield was calculated, according to the following equation:

Extraction yield (%) = m_dr_/m_pm_ × 100

where m_dr_ is the mass of the dry extract and m_pm_ is the mass of the plant material used for the extraction. The obtained dry residue was used to prepare a stock aqueous solution for further analysis.

### 2.3. Determination of the Content of Total Phenols

A 0.1% aqueous extract was used to quantify the phenol content using the Folin–Ciocalteu reagent method. An aqueous solution of Na_2_CO_3_ (65 g/L) and a 0.2 M solution of Folin–Ciocalteu reagent were used. After incubation, the absorbance was measured at 760 nm using distilled water as a blank. Measurements were performed in triplicate. The phenolic content was determined using a gallic acid calibration curve and expressed as milligrams of gallic acid equivalent per gram of dry extract (mg GAE/g d.e.) [20,21].

### 2.4. Determination of the Content of Total Flavonoids

Total flavonoid content was determined based on the ability of flavonoids to form a yellow-colored complex with aluminum ions. The Al reagent and distilled water were added to a 1% aqueous extract. After incubation, the absorbance was measured at 430 nm using distilled water as a blank. All measurements were conducted in triplicate. Flavonoid content was quantified using a quercetin calibration curve and expressed as milligrams of quercetin equivalents per gram of dry extract (mg QE/g d. e.) [20,21].

### 2.5. Chemical Characterization by High-Performance Liquid Chromatography

High-performance liquid chromatography (HPLC) coupled with a diode-array detector (DAD) was used to identify and quantify flavonoid and phenolic compounds, according to the previously described and validated analytical method developed by our research group [22,23]. The analysis was carried out using an Agilent HP 1100 HPLC-DAD system with an autosampler (Agilent Technologies, Waldbronn, Germany). The analyzed dry extract was dissolved in methanol prior to injection. Separation was achieved on a Nucleosil C18 column (4.6 mm × 250 mm, 5 μm) maintained at 30 °C, with an injection volume of 3 μL. The mobile phase consisted of formic acid in water (1%, *v*/*v*) as phase A and methanol as phase B with the gradient elution according to the scheme: 0–10 min, 10–25% B; 10–20 min, 25–45% B; 20–30 min, 45% B; 30–35 min, 45–70% B; 35–40 min, 70–100% B, 40–43 min, 100% B; 43–46 min, 100–10% B; 48 min, 10% B. The flow rate was varied as described in the original method [23]. The chromatograms were monitored at 280 nm for gallic, caffeic, and trans-cinnamic acids, 330 nm for *p*-coumaric, chlorogenic, rosmarinic and ferulic acids, and quercetin, and 350 nm for rutin and quercitrin. The concentrations of the individual compounds were expressed as µg/g of d.e.

### 2.6. Antioxidant Activity

The antioxidant activity of the *Artemisia annua* ethanolic extract was assessed based on its ability to scavenge 2,2-diphenyl-1-picrylhydrazyl (DPPH) and hydroxyl (OH) nitroso (NO) radicals, as well as its capacity to inhibit lipid peroxidation. Analyzed extract was assayed in various concentrations. The DPPH assay was determined at 515 nm. In the OH assay, free radicals were generated via the Fenton reaction, and the degradation of 2-deoxy-D-ribose to malonyldialdehyde (MDA) was monitored at 532 nm. The inhibition of the lipid peroxidation was evaluated based on the potential of the examined extract to protect the liposomes from free radicals formed in the Fenton reaction. The content of formed MDA was determined at 532 nm. In the NO radical inhibition assay, the donor of NO radicals was sodium nitroprusside, and the appearance of the purple complex with Greiss reagent was detected at 546 nm. All antioxidant assays were performed according to previously described methodologies [24,25]. The results were expressed as IC_50_ values representing the concentration required to neutralize 50% of DPPH, OH, and NO radicals, or to inhibit 50% of lipid peroxidation. Furthermore, the ferric reducing antioxidant power (FRAP) was assessed at three concentration levels according to a modified Benzie and Strain method [22,26]. Using the standard curve for ascorbic acid (AAE), the concentrations were calculated and expressed as milligrams of ascorbic acid equivalents per g of dry extract (mg AAEs/g d.e.).

### 2.7. Cytotoxic Activity

#### 2.7.1. Cell Culture

The rat pancreatic insulinoma cell line (Rin-5F) (ATCC^®^-CRL-2058™) was maintained in Roswell Park Memorial Institute (RPMI) medium (Sigma, St. Louis, MO, USA). The rat hepatoma cell line (H4IIE) (ATCC^®^ CRL-1548™) was grown in Minimum Essential Medium (MEM) (Sigma, St. Louis, MO, USA) while the human hepatocellular carcinoma cell line Hep G2 (ATCC^®^ HB-8066™) was cultured in Dulbecco′s Modified Eagle′s low-glucose medium (DMEM-LG) (Sigma, St. Louis, MO, USA). All media were supplemented with 10% fetal bovine serum (FBS), 1% L-glutamine, and 1% penicillin/streptomycin. Cells were cultured as adherent monolayers in a humidified incubator with 5% CO_2_ at 37 °C. Subculturing was performed when the cells reached 70–80% confluence. All reagents were purchased from Capricorn Scientific GmbH (Ebsdorfergrund, Germany).

#### 2.7.2. Cell Viability Assay

The cytotoxic effects of the *Artemisia annua* extract were assessed using the MTT assay, following a previously described protocol. Cells in the exponential growth stage were seeded in 96-well plates at a density of 10 000 cells per well for H4IIE and HepG2 cells, and 20 000 cells per well for Rin-5F cells. Due to its hydrophilic nature, the dry extract was resuspended in an appropriate medium for cell harvesting prior to the treatment. Cells were treated with the extract concentrations ranging from 0 to 300 µg/mL for 24 h. The cells treated only with a medium were used as the control. Following treatment, 0.5 mg/mL of MTT reagent (Sigma, St. Louis, MO, USA) was added to each well and incubated. A spectrophotometer plate reader (Multiscan MCC340, Labsystems, Vantaa, Finland) was used to measure the optical density (OD) at 540/690 nm. Cell viability of treated cells in comparison to the control cells (untreated 100%) was calculated according to the formula:

cell viability (%) = OD_test_/OD_control_ × 100

Two independent experiments were conducted, each in quadruplicate [27].

### 2.8. Statistical Data Processing

All data obtained from the assays of the analyzed *Artemisia annua* extract were processed using the software package Microsoft Excel for Windows (v. 2016). The MTT assay results were statistically analyzed using IBM SPSS version 26 (Chicago, IL, USA). Data were evaluated using one-way analysis of variance (ANOVA) followed by the Tukey post-hoc test. A *p*-value of less than 0.05 was considered statistically significant.

## 3. Results

### 3.1. Extraction Yield, Total Phenolic Content, and Total Flavonoid Content

The results of preliminary chemical characterization of the *Artemisia annua* ethanolic extract are summarized in Table 1.

In our study, the extraction yield was 14.53% using 70% ethanol as a solvent. This yield is higher than the previously reported values obtained using the same solvent and cold maceration method for 24 h but with a different drug-to-solvent ratio (1:20) [13]. On the other hand, Sembiring et al. obtained a 26.1% extraction yield with 70% ethanol, but using a somewhat different extraction procedure and drug-to-solvent ratio (1:5) [28]. The analyzed plant material was rich in phenolic compounds, namely, 66.74 mg GAE/g d.e. for total phenolics and 12.57 mg QE/g d.e. for total flavonoids. These concentrations are higher when compared to other studies conducted with ethanolic extracts for both total phenolics and total flavonoids [13,29].

### 3.2. Chemical Characterization by High-Performance Liquid Chromatography (HPLC)

**Figure 2:**
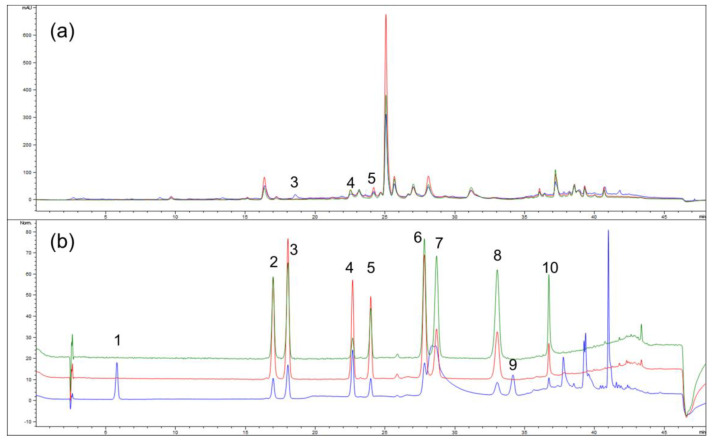
HPLC-DAD chromatogram of (**a**) *Artemisia annua* extract and (**b**) calibration standards mixture solution with detection at 280 nm (blue), 330 nm (red), and 350 nm (green): 1—gallic acid, 2-p—coumaric acid, 3—quercetin, 4—caffeic acid, 5—chlorogenic acid, 6—rosmarinic acid, 7—rutin, 8—quercitrin, 9—trans–cinnamic acid, 10—ferulic acid.

The HPLC-DAD analysis of the *Artemisia annua* extract revealed the presence of caffeic acid, chlorogenic acid, and quercetin (Table 2, Figure 2). Among the identified compounds, chlorogenic acid was found in the highest concentration, indicating that the extract is a particularly rich source of this phenolic acid. Caffeic acid was also detected, albeit at a lower concentration. The quercetin content was notably lower in the analyzed sample. Other investigated phenolic and flavonoid compounds were not detected under the applied chromatographic conditions. One study separated and quantified two hydroxycinnamic acids, caffeic and chlorogenic acid using high-performance liquid chromatography, coupled with mass spectrometry, and separated using a Zorbak SB-C18 reverse phase analytical column. The chromatography method, the column used, and the detection applied in that study differ from those employed in our research which may account for the discrepancies in the results. The content of caffeic acid was approximately four times lower (50.20 µg/g of d.e.), while the concentration of chlorogenic acid was markedly higher (4658.60 µg/g of d.e.) compared to our findings [30]. Also, Bernatoniene et al. determined the concentration of caffeic and chlorogenic acids in *Artemisia annua* hydroalcoholic extracts. However, the extraction procedure was not explained with enough detail to compare the results with our study [31].

### 3.3. Antioxidant Activity

Five different in vitro assays were used for the assessment of the antioxidant activity of *Artemisia annua* ethanolic extract. The results are summarized in Table 3. The extract showed notable free radical scavenging potential, particularly against the synthetic radical 2,2-diphenyl-1-picrylhydrazil (DPPH) and the biologically occurring hydroxyl radical (OH^•^). Sembiring et al. reported that *Artemisia annua* ethanolic extract exhibited slightly lower potency in scavenging DPPH radicals compared to our results (5.17 μg/mL) [28]. Additionally, in another study conducted on *Artemisia annua* from France, 0.5 mg/mL ethanolic extract achieved 64.13% inhibition of DPPH radicals [32]. One study revealed a lower DPPH radical scavenging activity of the *Artemisia annua* methanolic extract (IC_50_ = 77.73 ± 1.15 μg/mL), compared to our findings [30].

In our study, the ethanolic extract of *Artemisia annua* showed strong efficacy in scavenging hydroxyl radical (OH^•^), achieving 50% inhibition at a relatively low concentration (IC_50_ = 17.8 µg/mL). The tested extract proved to be less effective in targeting nitric oxide radicals (NO^•^) and inhibiting the lipid peroxidation, with 50% inhibition requiring a higher concentration (IC_50_ = 79.94 µg/mL and IC_50_ = 41.56 µg/mL, respectively). Nevertheless, in one study, the 95% ethanol extract of *Artemisia annua,* prepared with a different extraction procedure, strongly inhibited the NO radical production in macrophage cells. The extract was tested in the concentrations ranging from 6.25 to 50 µg/mL, with inhibition values ranging from 72.39 ± 4.94 to 104.16 ± 1.08% [33]. The results of the FRAP assay were in accordance with previously conducted investigations taking into account that a wide range of variation in the expression of FRAP values has been reported in the literature [13].

These findings suggest that the antioxidant capacity of *Artemisia annua* extract is primarily mediated through radical scavenging mechanisms, especially for highly reactive oxygen species.

### 3.4. Cytotoxic Activity

**Figure 3:**
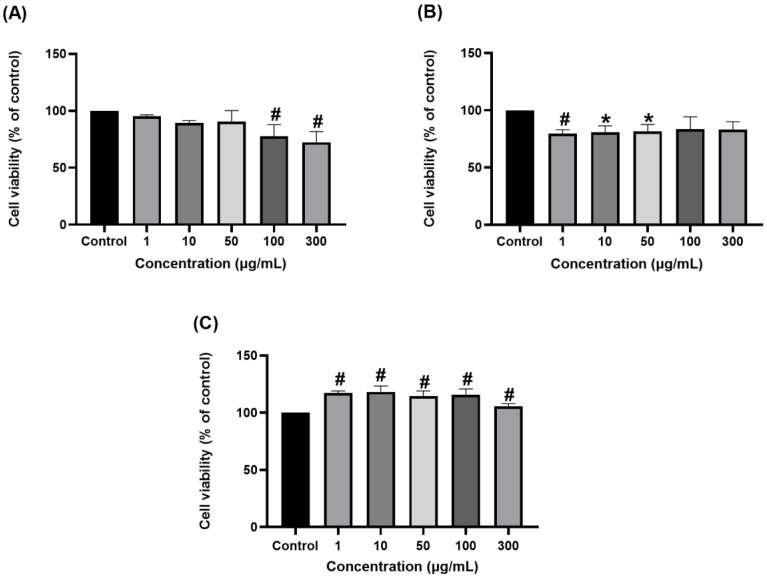
Effects of different concentrations of *Artemisia annua* extract in the concentration range 0–300 µg/mL, on Rin5F (**A**), HepG2 (**B**), and H4IIE (**C**) cell viability after 24 h of the treatment. Data are presented as mean  ±  S.D. * *p* < 0.05; # *p* < 0.01.

The results of the MTT cell viability test are presented in Figure 3. The *Artemisia annua* extract exhibited a concentration-dependent inhibitory effect on Rin5F pancreatic insulinoma cells (Figure 3A), indicating potential antiproliferative activity. In HepG2 human hepatocellular carcinoma cells (Figure 3B), the extract induced a moderate but consistent reduction in cell viability across the tested concentration range. Conversely, in H4IIE rat hepatoma cells (Figure 3C), certain concentrations of the extract appeared to enhance cell viability, suggesting a potential hormetic or a cell-specific adaptive response.

Previous studies have also demonstrated the significant anticancer potential of *Artemisia annua,* with documented cytotoxic effects against various cancer cell lines, including human digestive tumor, liver carcinoma (HepG2), and gastric cancer cells. In addition, the *Artemisia annua* extract mitigated the hepatotoxicity induced by doxorubicin. These findings support the notion that *Artemisia annua* may serve as a promising chemopreventive agent [2,34,35,36].

## 4. Discussion

*Artemisia annua* extracts are a valuable resource of different pharmacologically active compounds with the promising antimalarial, antioxidant, anti-inflammatory, anticancerogenic, antiviral, and antiparasitic actions. Based on the results of our study and compared to other research, it can be discerned that the extraction yield of various *Artemisia annua* extracts depended on the solvent concentration and polarity as well as the extraction procedure and drug-to-solvent ratio [13,28].

Undoubtedly, the analyzed extract is a rich source of phenolic compounds. The differences in the TP and TF contents between studies examining ethanolic extracts can be partially explained by variations in the extraction procedures, as well as differences in the geographical origin of the plant material, climate, growth, and cultivation conditions, and the part of the plant used [37]. Skowyra et al. examined a greenhouse-grown ethanolic extract of the *Artemisia annua* leaves and reported significantly lower concentrations of TF (2.68 ± 0.07 mg of catechin equivalents per gram of d.e.) [29]. The main distinction between the described extract and the one used in our study stems from the usage of different parts of *Artemisia annua* for extract preparation. The herb consists of leaves, stems, and inflorescence, the latter being yellow in color. It is possible that the inflorescence contributes to the higher phenolic content, particularly as these compounds are known yellow pigments typically present in flowers; where they serve as attractants for pollinators and prove protection against UV radiation [38]. Additionally, several studies have examined the influence of solvent type on the TP and TF content in *Artemisia annua* extracts. Iqbal et al. recorded that methanol was the most effective solvent for extracting phenolic compounds from *Artemisia annua* leaves [39]; unfortunately, ethanol was not included in this study for comparison. Nevertheless, ethanol was selected in our investigation, due to its lower toxicity, greater availability, and the polarity comparable to that of methanol.

Numerous scientific papers have dealt with the HPLC analysis of the phenolic compounds in different *Artemisia annua* extracts. Dilshad et al. identified four out of six phenolic markers (gallic acid, catechin, rutin, quercetin, isoquercetin, and caffeic acid) studied by HPLC in the methanolic extract of the wild type *Artemisia annua.* These include rutin, isoquercetin, and, as in our extract, quercetin (10 µg /g d.e.) and caffeic acid (30 µg/g d.e.). In our extract, quercetin (36.18 µg/g d.e.) and caffeic acid (202.41 µg/g d.e.) were present in significantly higher concentrations [40]. However, Dilshad et al. used 6-months old shoots for the extract preparation. These differences likely reflect variations in the plant parts used for the extraction, reinforcing the importance of the harvesting time of the plant material [40]. The reverse phase HPLC-DAD method was also applied to quantify phenolic acids and polyphenolic compounds in the ethanolic extract of the *Artemisia annua* hairy roots cultivated in Ukraine. Compared to our results, the quercetin content was approximately the same, while the chlorogenic acid content varied substantially, underscoring the influence of both plant origin and the plant part used for extraction [37].

Polyphenolic compounds represent a widespread and functionally significant group of secondary plant metabolites. In recent years, these substances, especially flavonoids and phenolic acids, have attracted considerable scientific interest due to their pronounced antioxidant capacity, which contributes to their valuable therapeutic potential in the prevention of free radical-mediated diseases [30]. These compounds play an important role in maintaining cellular redox homeostasis and mitigating oxidative damage [37]. Numerous studies have proven a strong correlation between the antioxidant capacity of medicinal plants and their phenolic content [41]. In particular, caffeic and chlorogenic acids and flavonoids such as rutin, quercetin, luteolin, and apigenin, recently identified in many *Artemisia annua* cultivars, are considered potent antioxidants [42]. The DPPH radical scavenging assay is one of the preliminary assays performed in order to elucidate whether the investigated plant extract possess antioxidant potential. The antioxidant activity of different *Artemisia annua* extracts in the DPPH assay was studied, often being correlated with the total phenolic and flavonoid contents [30]. As in our study, the extract showed notable free radical scavenging potential against the synthetic DPPH^•^, but also against the biologically relevant and highly reactive hydroxyl radical (OH^•^). Additionally, researchers have compared the inhibition of lipid peroxidation among *Artemisia annua* leaf extracts prepared using different solvents. The highest inhibition of lipid peroxidation (24.24 ± 1.04%) was recorded in the chloroform extract, while the lowest (10.72 ± 0.07%) was observed in the methanol extract at the same tested concentration of 0.1 mg/mL. In our research, 50% inhibition of lipid peroxidation was achieved with an extract concentration of 41.56 µg/mL, indicating substantially higher antioxidant potency. Moreover, researchers have examined the potential of a crude ethanol extract of *Artemisia annua* to inhibit NO radical production in macrophage cells. The extract was tested in the concentrations ranging from 6.25 to 50 µg/mL, with the inhibition values ranging from 72.39 ± 4.94 to 104.16 ± 1.08% [33]. Such high values indicate a more pronounced antioxidant potential of the crude extract compared to our ethanolic extract, which at a higher concentration (79.94 µg/mL) demonstrated only 50% of NO^•^ inhibition. As observed in many investigations and corroborated by our findings, *Artemisia annua* extract exhibited a concentration-dependent inhibition of various reactive oxygen species and lipid peroxidation. Also, the findings reinforce the critical role of solvent selection in extraction efficiency and, consequently, in the antioxidant potential of plant-derived extracts [39]. However, more research is needed to fully elucidate the antioxidant capacity and underlying mechanisms of *Artemisia annua* extracts in mitigating oxidative stress.

In recent years, the use of in vitro cell culture models has gained substantial attention in biomedical research due to several key advantages. These models are cost effective, easily manipulated, and provide an unlimited supply of material. Perhaps most significantly, cell lines serve as established models in cancer biology research, facilitating the evaluation and optimization of anticancer therapies [43,44]. The cytotoxicity assays performed on cancer cell lines provide crucial preliminary data for identifying biologically active compounds with potential pharmacological applications.

The anticancer potential of various *Artemisia annua* extracts has not only been demonstrated in cell and animal models but has also been reported in human studies. One such case report described the use of *Artemisia annua* capsules in a patient diagnosed with prostate carcinoma. Long-term administration of the capsules resulted in a marked decrease in prostate-specific antigen (PSA) levels, a key tumor marker for prostate cancer. However, after seven months of use, the tumor sensitivity to *Artemisia annua* decreased. Since this case report involved only a single patient, its findings are anecdotal, and well-designed clinical trials are essential to rigorously evaluate the therapeutic efficacy and safety of *Artemisia annua* in cancer patients [45].

In the present study, we evaluated the cytotoxic effects of *Artemisia annua* ethanolic extract on the rat pancreatic insulinoma cell line (Rin-5F), the rat hepatoma cell line (H4IIE), and the human hepatocellular carcinoma (HepG2). The extract exhibited an antiproliferative effect at all tested concentrations in the rat insulinoma and human hepatocarcinoma cells, while it improved the survival of rat hepatoma cells. However, based solely on the obtained results, it is difficult to provide a mechanistic explanation or identify the possible molecular pathways. To our knowledge, no previous studies have reported the effects of *Artemisia annua* on these specific cell lines. Still, related in vitro studies on other cancer cell lines have shown that hydroalcoholic extracts of *Artemisia annua* can induce cell cycle arrest at the S and G2/M phases in a dose-dependent manner. This is also accompanied by a loss of mitochondrial membrane potential, caspase 3 activation, and the formation of an apoptotic hypodiploid cell population [46,47]. Further research should focus on elucidating the molecular mechanisms and cellular pathways affected by *Artemisia annua* extracts, particularly those related to oxidative stress responses, gene expression regulation, and intracellular signaling cascades in both human and animal models. In addition, the observed differences in cytotoxic responses between HepG2 and H4IIE cells highlight species-specific variations and suggest that HepG2 may be a more reliable in vitro model for predicting human cellular responses to the chemical constituents of medicinal plants [48].

## 5. Conclusions

The results of this study confirm that *Artemisia annua* is a rich source of bioactive phenolic and flavonoid compounds, with phenolic acids—particularly chlorogenic and caffeic acid—being the most abundant. The ethanolic extract demonstrated notable antioxidant activity through the effective inhibition of oxidative stress mediators, especially free radicals. Among the tested radicals, the extract exhibited the highest neutralizing capacity against hydroxyl (OH^•^) and DPPH radicals. At moderately higher concentrations, it also showed the ability to scavenge nitric oxide (NO^•^) radicals and inhibit lipid peroxidation processes. Despite limitations such as a relatively small sample size and testing under specific experimental conditions, our findings suggest that *Artemisia annua* ethanolic extract may contribute to the reduction in oxidative stress in biological systems. This effect could potentially aid in the prevention or mitigation of oxidative stress-related diseases and their associated complications. Moreover, results from the MTT cell viability assay indicate that the extract possesses antiproliferative activity, particularly against the rat insulinoma (Rin-5F) and human hepatocellular carcinoma (HepG2) cell lines. These preliminary in vitro findings provide promising evidence of the therapeutic potential of *Artemisia annua*, warranting further investigation focused on its detailed cellular mechanisms.

## Figures and Tables

**Table 1:** Preliminary chemical characterization of *Artemisia annua* ethanolic extract.

Preliminary Test	Value (Mean ± SD)
Extraction yield (%)	14.53 ± 0.58
Content of total phenolics (mg GAE/g d.e.)	66.74 ± 5.03
Content of total flavonoids (mg QE/g d.e.)	12.57 ± 0.21

GAE—Gallic acid equivalent; QE—quercetin equivalent; d.e.—dry extract.

**Table 2:** Detailed chemical composition of *Artemisia annua* ethanolic extract (expressed as μg/g of d.e.; values reported as mean SD).

Phenolic Compound *	Caffeic Acid	Chlorogenic Acid	Quercetin
Structural formula	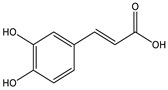	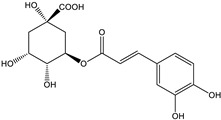	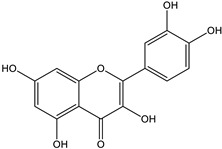
Content (μg/g d.e.)	202.41 ± 10.12	651.14 ± 32.56	36.18 ± 2.53

* Gallic acid, trans-cinnamic acid, p-coumaric acid, ferulic acid, rosmarinic acid, quercitrin, and rutin were below limit of detection of the applied analytical method.

**Table 3:** Antioxidant potential of *Artemisia annua* ethanolic extract (expressed as IC_50_ in μg/mL for DPPH, OH, NO, and LP tests and in mg AAE/g d.e. for FRAP test).

Assay	DPPH	OH^•^	NO^•^	LP	FRAP
Extract	5.17 ± 0.71 ^a^	17.83 ± 1.23 ^a^	79.94 ± 2.84 ^a^	41.56 ± 2.73 ^a^	24.62 ± 5.27
PG	0.61 ± 0.07 ^b^	9.51 ± 0.75 ^b^	9.23 ± 0.39 ^b^	/	/
BHT	/	0.05 ± 0.01 ^c^	/	8.45 ± 0.55 ^b^	/
AA	/	2.09 ± 0.11 ^d^	/	/	/

DPPH—2,2-diphenyl-1-picrylhydrazyl; OH^•^—hydroxyl radicals; NO^•^—nitroso radicals; LP—lipid peroxidation; PG—propyl gallate; BHT—butylated hydroxytoluene; AA—ascorbic acid. Different lower-case letters indicate statistically significant differences (*p* < 0.05).

## Data Availability

Data is contained within the article.
